# Targeted alpha therapy of mCRPC: Dosimetry estimate of ^213^Bismuth-PSMA-617

**DOI:** 10.1007/s00259-017-3817-y

**Published:** 2017-09-11

**Authors:** Clemens Kratochwil, Karl Schmidt, Ali Afshar-Oromieh, Frank Bruchertseifer, Hendrik Rathke, Alfred Morgenstern, Uwe Haberkorn, Frederik L. Giesel

**Affiliations:** 1 Department of Nuclear Medicine, University Hospital Heidelberg, INF 400, 69120 Heidelberg, Germany; 2 ABX-CRO, Dresden, Germany; 3 Directorate for Nuclear Safety and Security, European Commission - Joint Research Centre, Karlsruhe, Germany; 4 Cooperation Unit Nuclear Medicine, German Cancer Research Center (DKFZ), Heidelberg, Germany

**Keywords:** Bi-213, Targeting alpha therapy, PSMA, Prostate cancer

## Abstract

**Purpose:**

PSMA-617 is a small molecule targeting the prostate-specific membrane antigen (PSMA). In this work, we estimate the radiation dosimetry for this ligand labeled with the alpha-emitter ^213^Bi.

**Methods:**

Three patients with metastatic prostate cancer underwent PET scans 0.1 h, 1 h, 2 h, 3 h, 4 h and 5 h after injection of ^68^Ga-PSMA-617. Source organs were kidneys, liver, spleen, salivary glands, bladder, red marrow and representative tumor lesions. The imaging nuclide ^68^Ga was extrapolated to the half-life of ^213^Bi. The residence times of ^213^Bi were forwarded to the instable daughter nuclides. OLINDA was used for dosimetry calculation. Results are discussed in comparison to literature data for ^225^Ac-PSMA-617.

**Results:**

Assuming a relative biological effectiveness of 5 for alpha radiation, the dosimetry estimate revealed equivalent doses of mean 8.1 Sv _RBE5_/GBq for salivary glands, 8.1 Sv _RBE5_/GBq for kidneys and 0.52 Sv _RBE5_/GBq for red marrow. Liver (1.2 Sv _RBE5_/GBq), spleen (1.4 Sv _RBE5_/GBq), bladder (0.28 Sv _RBE5_/GBq) and other organs (0.26 Sv_RBE5_/GBq) were not dose-limiting. The effective dose is 0.56 Sv _RBE5_/GBq. Tumor lesions were in the range 3.2–9.0 Sv_RBE5_/GBq (median 7.6 Sv_RBE5_/GBq). Kidneys would limit the cumulative treatment activity to 3.7 GBq; red marrow might limit the maximum single fraction to 2 GBq. Despite promising results, the therapeutic index was inferior compared to ^225^Ac-PSMA-617.

**Conclusions:**

Dosimetry of ^213^Bi-PSMA-617 is in a range traditionally considered reasonable for clinical application. Nevertheless, compared to ^225^Ac-PSMA-617, it suffers from higher perfusion-dependent off-target radiation and a longer biological half-life of PSMA-617 in dose-limiting organs than the physical half-life of ^213^Bi, rendering this nuclide as a second choice radiolabel for targeted alpha therapy of prostate cancer.

**Electronic supplementary material:**

The online version of this article (10.1007/s00259-017-3817-y) contains supplementary material, which is available to authorized users.

## Introduction

A survival benefit observed in bone metastatic prostate cancer after alpha radiation therapy with the bone-seeking ^223^RaCl_2_, which could not be demonstrated for its beta-emitting analogue ^89^SrCl_2_, increased the interest in alpha-emitter-based radionuclide therapy [1]. However, ^223^RaCl_2_ accumulates in the bone remodeling zone of metastases and hits tumor cells only indirectly and insufficiently; extra-osseous lesions are not treated at all. PSMA-617 is a small molecule targeting the prostate-specific membrane antigen (PSMA), which is directly expressed by the tumor cells [2]. PSMA-targeting radio-ligand therapy (RLT) based on the beta-emitting ^177^Lu-PSMA-617 has been reported as being well-tolerated and presents promising anti-tumor activity [3]. More recently, initial results with targeting alpha therapy (TAT) based on ^225^Ac-PSMA-617 imply even higher anti-tumor activity and less hematological toxicity in case of diffuse red marrow infiltration [4, 5]. ^213^Bi is another alpha particle-emitting nuclide already used in clinical application [6–8]. Tagged to the PSMA-mAB J591, ^213^Bi demonstrated promising efficacy in particular against prostate cancer in the preclinical setting [9, 10]. In comparison to ^225^Ac (T_1/2_ 9.9d), due to its short physical half-life, rapid tumor targeting is more pivotal with ^213^Bi (T_1/2_ 46 min). However, the low-molecular-weight PSMA-617 demonstrates fast pharmacokinetics in both animals [2] and human beings [11], and led to the discussion whether ^213^Bi can serve as an alternative to ^225^Ac for PSMA-TAT. Recently, a single case of promising anti-tumor activity of ^213^Bi-PSMA-617 was reported [12]. Using ^68^Ga (T_1/2_ 68 min) as a surrogate nuclide enabling high-resolution quantitative ^68^Ga-PSMA-617 PET-imaging, we estimated the extrapolated radiation dosimetry for ^213^Bi-PSMA-617 and compared its therapeutic index with findings for ^225^Ac-PSMA-617.

## Methods

### Patients

The used imaging data sets have already been used to calculate the dosimetry estimate of ^68^Ga-PSMA-617 as a diagnostic agent as part of an evaluation approved by the local ethics committee and including written informed consent [11]. These anonymized data were evaluated for extrapolation of dosimetry to ^213^Bi. No additional experiments involving human beings were required for this work.

Detailed patients characteristics are provided in the supplement (Supplement/Table 1). All study subjects had a moderate total tumor volume; creatinine values were in the normal range, respectively. Thus, our results may not apply for patients with underlying kidney disease or excessive tumor burden.

### Imaging

Exams were performed in a BIOGRAPH-mCT PET/CT scanner (Siemens). An unenhanced low-dose CT scan [120 keV/60mAs at 3 h and 80 keV/30mAs at other time points; 5-mm slice thickness, 2-mm increment, B31 (Siemens) soft-tissue reconstruction kernel] was obtained at 0.1 h, 1 h, 2 h, 3 h, 4 h and 5 h after injection of 213–260 MBq ^68^Ga-PSMA-617 immediately followed by a whole-body (vertex to upper legs) PET in 3-dimensional mode (matrix 200 × 200). Each bed position (axial field of view, 16.2 cm) was acquired for 3 min. The emission data were corrected for random, scatter and attenuation. Reconstruction was conducted with an ordered-subset expectation maximization algorithm with two iterations and eight subsets and Gauss-filtered with 2 mm in full width at half maximum. Urine was collected before each PET/CT measurement and venous blood samples were taken. For illustrative purposes, the decay-corrected PET images, presented in linear gray scale and normalized to the time point 2 h p.i., were used to calculate quantitatively correct shadings taking into account the physical half-life of ^213^Bi and ^225^Ac.

### Dosimetry calculation

The dosimetry analysis was performed using the QDOSE dosimetry software suite (ABX-CRO Advanced Pharmaceutical Services, Dresden, Germany). All six imaging time points were coregistered with automatic rigid and, if applicable, deformable coregistration of CT images. PET scans were coregistered by coupling to the transformation matrix of the coupled CT. Segmentation was done by a radiologist, who defined the organ edges on the CT images. To simplify the drawing process, preliminary volumes of interest (VOIs) for kidneys, liver, spleen, parotid glands, submandibular glands and urinary bladder were first drawn manually and then the “percent of maximum threshold” of an automatically adapted VOI in the corresponding PET images was varied, until the VOI matched the morphological delineated organ contour. The VOIs were then copied onto all other time points to generate the time-activity curves (TACs) for all segmented organs, respectively. A VOI covering the complete field of view was used as a surrogate to calculate the effective half-life in the total body, assuming that the distant limbs (cropped during PET/CT imaging) behave similarly as the trunk-torso. This effective half-life was then applied to the injected activity to derive the total body TAC. The TACs for red marrow were based on venous blood samples following established models [13, 14], excluding patient 1 due to missing values caused by poor vein conditions. The urinary bladder content TAC was a combination of estimated activity in the urinary bladder content VOI in the PET images and measured activity of five collections of voided urine. The activity of ^68^Ga at each TAC time point was corrected for physical decay (T1/2 68 min) since time of injection. Then, the decay formula was applied vice versa with T1/2 46 min from time of injection to the respective time point to calculate the activity of ^213^Bi. Thus, TACs intended to represent ^213^Bi-PSMA-617 were calculated, taking into account the different half-life of the modeled radionuclide but assuming that the nuclide replacement does not affect the pharmacokinetics of the shuttle molecule. Red marrow, total body, urinary bladder content and all segmented organ TACs (Supplement/Table 2) were loaded into QDOSE to be considered in further calculations. Curve fitting was then applied to all organ TACs. For all cases, a mono-exponential curve fit was used. If possible, all time points or time points 2 to 6 were used for the curve fit. In salivary glands, lesions and kidneys, an increasing uptake during the first three time points was observed, leading to an effective half-life greater than the physical half-life. In those cases, only the last three to six time points were used for curve fitting. Activity integration was done assuming a linear increase from 0 to the first measured activity, using trapezoidal approximation between the first and last measured time point and from the last measured time point to infinity using the fitted function. The remainder body was calculated by subtracting all source organs from total body. Residence times were calculated by dividing the cumulated activity with the injected activity. The ^213^Bi residence times of all included source organs and remainder body were used for equivalent dose calculation in OLINDA [15] using the original organ masses of the “adult male” phantom, as only selective patients without morphological abnormalities have been chosen for this modeling. The doses absorbed to the salivary glands and tumor lesions were determined using the spherical model [16].

**Fig. 1:**
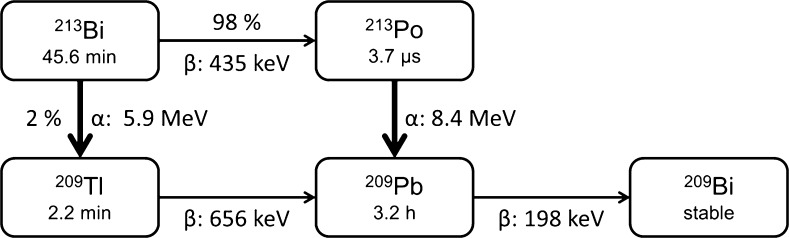
Decay schema of ^213^Bi

The same residence time of ^213^Bi was used to calculate equivalent doses for all daughter nuclides (^209^Tl, ^213^Po and ^209^Pb) in OLINDA. The finally absorbed and effective doses are the sum of these nuclides, weighting ^209^Tl with 2% and ^213^Po with 98%, following the decay scheme of ^213^Bi (Fig. 1) [25]. For ^209^Pb, 100% of the initial ^213^Bi residence time was used again. We assumed instant decay, i.e. a possible translocation of daughter nuclides between the succeeding disintegrations was neglected, which can be justified due to the short 3.7-μs half-life of ^213^Po in the main decay branch and the limited contribution of the low-energy beta-emitter ^209^Pb to the summed absorbed dose.

The calculated absorbed doses were translated into equivalent doses using weighting factors of 5 for alpha and 1 for beta−/gamma radiation, from now on, referred to as “Sv_RBE5_“. The concept of the relative biological effectiveness (RBE) of alpha radiation with regard to deterministic effects was reviewed in detail by the US Department of Energy [17] and the Committee on Medical Internal Radiation Dose [18], which came to the conclusion that an RBE = 5 presents the most reasonable assumption for the 8.4 MeV alpha particle energy predominantly emitted during decay of ^213^Bi. The uncertainties within this concept have already been discussed with regard to ^225^Ac-PSMA-617 [5]. However, the consistent application of model assumptions is helpful to improve the comparability with the literature values for the ^225^Ac-PSMA-617 bench mark [5].

## Results

Interim results, such as residence time, biological and effective half-life, are provided in the Supplement/Table 3. The dose approximation from the last measured time point to infinity contributed <5% to the cumulated activity, putting aside the need for more sophisticated techniques than mono-exponential curve fitting. The finally calculated safety dosimetry for normal organ radiation exposure in equivalent doses is presented in Table 1. The dose contributions for the respective decay steps, as illustrated in Fig. 1, are presented in detail within the Supplement/Table 4.

**Table 1:** Projected organ equivalent doses for ^213^Bi-PSMA-617 (Sv_RBE5_/GBq, assuming RBE = 5 for alpha and RBE = 1 for beta and photon radiation)

Organ	Equivalent dose (Sv_RBE5_/GBq, mean ± SD)	
Adrenals	0.27	±0.02
Brain	0.27	±0.02
Breasts	0.27	±0.02
Gallbladder wall	0.27	±0.02
LLI wall	0.27	±0.02
Small intestine	0.27	±0.02
Stomach wall	0.27	±0.02
ULI wall	0.27	±0.02
Heart wall	0.27	±0.02
Kidneys	8.11	±1.80
Liver	1.20	±0.12
Lungs	0.27	±0.02
Muscle	0.27	±0.02
Ovaries	0.27	±0.02
Pancreas	0.27	±0.02
Red marrow	0.52	±0.15
Osteogenic cells	3.55	±0.77
Skin	0.27	±0.02
Spleen	1.42	±0.79
Testes	0.27	±0.02
Thymus	0.27	±0.02
Thyroid	0.27	±0.02
Urinary bladder wall	0.28	±0.03
Uterus	0.27	±0.02
Total body	0.34	±0.02
Effective dose equivalent	0.99	±0.09
Effective dose	0.56	±0.07

Projected equivalent doses for salivary glands (which is not provided by OLINDA because they have not been considered in the Cristy–Eckerman phantom) and mean values for estimated tumor equivalent doses were calculated using the spherical model (interim results presented in the Supplement/Table 5). They are presented in Table 2, along with a brief comparison to our results for dosimetry modeling of ^225^Ac-PSMA-617, which was based on surrogate imaging using ^177^Lu-PSMA-617 [5, 19], and is also highlighting the most dose-critical organs.

**Table 2:** Equivalent doses for critical organs and tumor lesions of PSMA-617 tagged with various radiolabel nuclides (^225^Ac per MBq, ^213^Bi and ^177^Lu per GBq) assuming homogenous dose distribution

	^213^Bi-PSMA-617 [Sv_RBE5_/GBq]	^213^Bi-PSMA-617 [Sv_RBE5_/1.2GBq]	^225^Ac-PSMA-617 [Sv_RBE5_/MBq]	^225^Ac-PSMA-617 [Sv_RBE5_/ 7.4 MBq]	^177^Lu-PSMA-617 [Gy/GBq]	^177^Lu-PSMA-617 [Gy/ 7.4 GBq]
	This work		Ref. [5]		Ref.[19]	
Salivary Gl.	8.1	9.7	2.3	17.0	1.4	10.4
Kidneys	8.1	9.7	0.7	5.2	0.8	5.9
Red marrow	0.52	0.6	0.05	0.4	0.03	0.2
Tumor lesions	6.3 (3.2–9.0)	7.6	5.7 (1.3–9.8)	42.1	6.6 (6–22)	48.8

The equivalent doses in Sv_RBE5_ as provided in Table 1 and Table 2 were calculated assuming a relative biological effectivity of 5 for alpha and 1 for beta and gamma radiation. Using these weightings, the contribution of alpha, beta and photon radiation is mean 98.3%, 1.5% and 0.2%, respectively, for ^213^Bi, compared to 99.4% alpha, 0.5% beta and 0.1% photon radiation already reported for ^225^Ac [5].

Consisting mainly of alpha and short-range beta radiation, the absorbed dose of a particular OLINDA source organ can be considered almost directly proportional to the inverse of its mass. As provided in the Supplement/Table 1, the body weights of patients 2 and 3 (70 and 72 kg, respectively) are close to the male adult phantom (74 kg); however, patient 1 (110 kg) causes a mentionable overestimation of whole-body remainder organs. For kidney masses, the situation is similar; here patients 1 and 2 are well in line with the reference man; but in patient 3, the kidney dose should be overestimated. Neglecting individual organ masses, we have to consider a methodical uncertainty of approx. 20% for the average values of our patients. However, dose-limiting organs tend to be overestimated, which is considered the preferred bias with regard to a conservative safety dosimetry.

Assuming an acute tolerance limit of 1 Sv_RBE5_ for red marrow, a cumulative equivalent dose of 27 Sv_RBE5_ for kidneys and 17 Sv_RBE5_ per cycle to salivary glands [5], the maximum tolerable single fraction of ^213^Bi-PSMA-617 could be projected to an activity of about 2 GBq per cycle and a cumulative treatment activity to approx. 3.6 GBq. To exploit the maximum possible cumulative dose while simultaneously keeping safety margins of the maximum single dose, fractionation regimens of, e.g. 2 × 1.8 GBq, 3 × 1.2 GBq or 4 × 0.9 GBq, might be necessary.

**Fig. 2:**
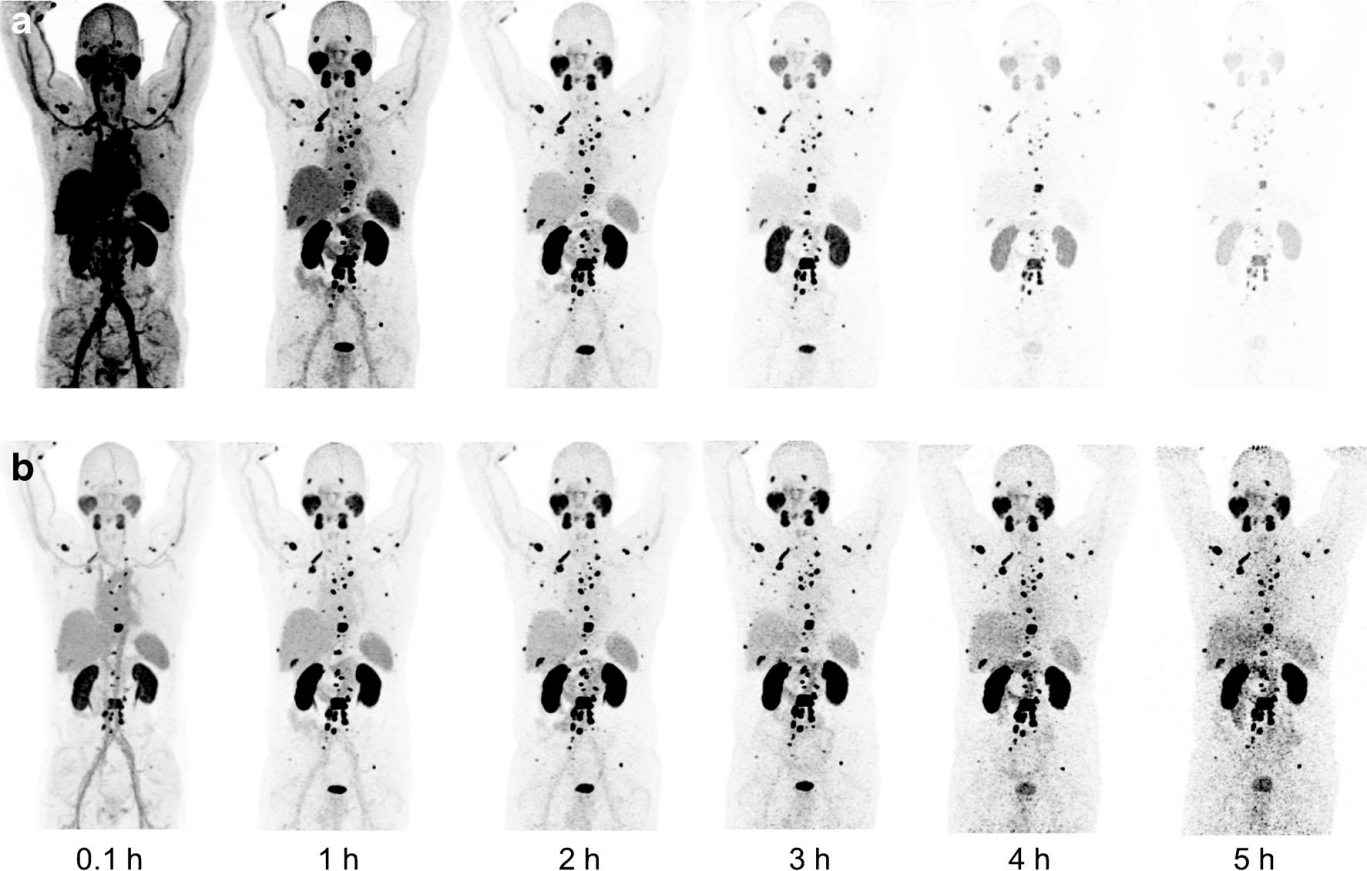
Visualization of the different nuclide distribution taking into account the half-life of ^213^Bi (**a**) or ^225^Ac (**b**). Maximum intensity projections of ^68^Ga-PSMA-617 PET scans, translated into quantitatively correct shadings using a linear gray scale normalized to time point “2 h p.i.”

**Fig. 3:**
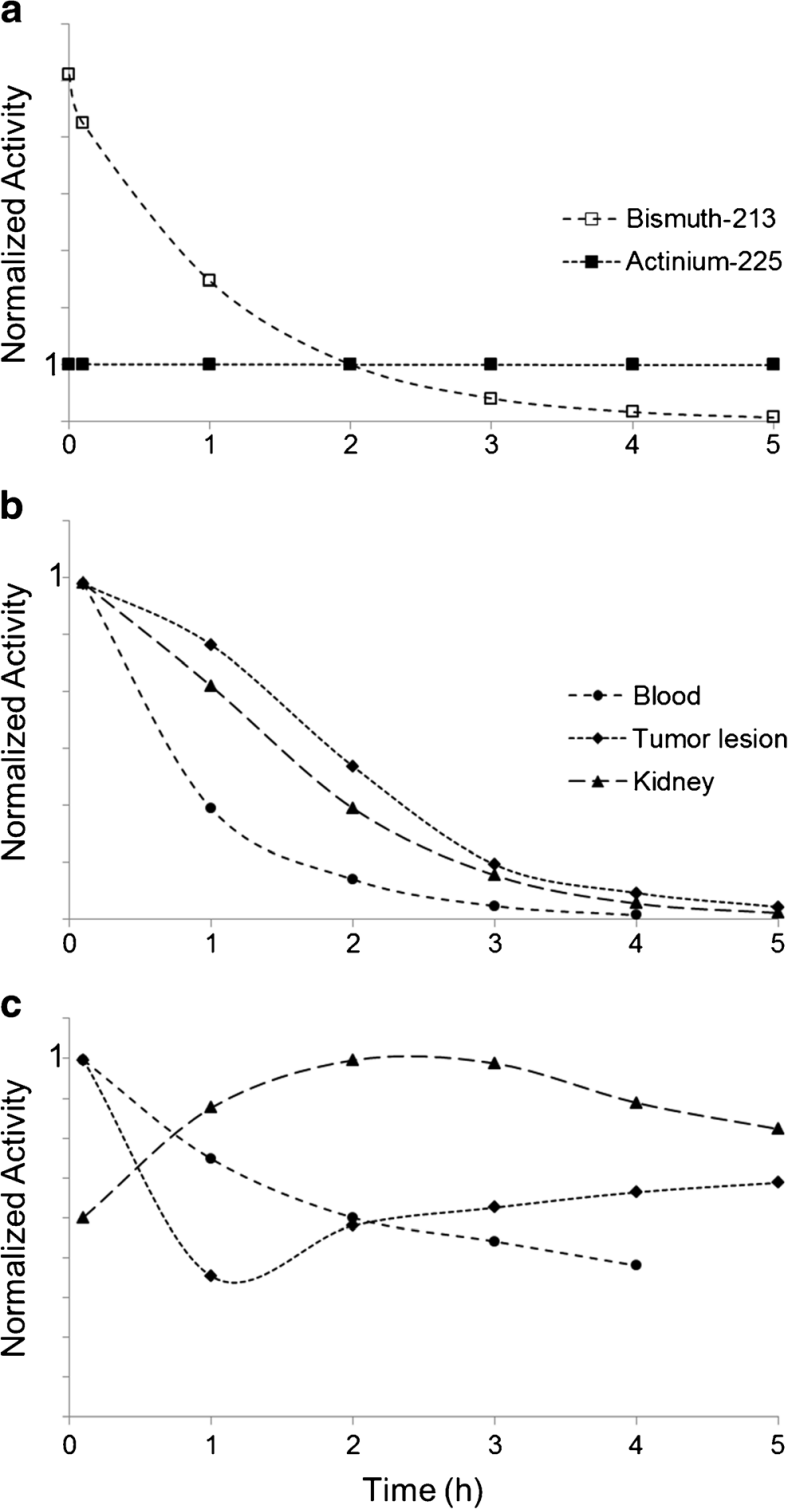
Physical decay of ^213^Bi and ^225^Ac, normalized to the time point “2 h” (**a**). For ^213^Bi-PSMA-617, the time-activity curves of all organs are dominated by the physical half-life of the nuclide (**b**). For ^225^Ac PSMA-617, the corresponding time-activity curves of are more affected by the pharmacokinetics of the ligand (**c**). Subparts (b) and (c) share the same legend; one representative “tumor lesion” and sample organs were chosen by chance from the patient presented in Fig. 2. Curves are normalized to their respective maximum value

In comparison to ^225^Ac-PSMA-617, the ratios between tumor and all potentially dose-limiting organs are lower. The calculated results are illustrated with the maximum intensity projections of the serially performed PET scans taking into account the physical decay (Fig. 2): After 5 h, ^213^Bi (T_1/2_ 46 min) already decayed by 98.9%, and ^225^Ac (T_1/2_ 9.9 d) only decayed by 1.5%. Time-activity curves demonstrate how the half-life of the radiolabel and the pharmacokinetics of the shuttle molecule contribute to the tissue activity concentrations over time (Fig. 3).

## Discussion

The aim of this work was to estimate radiation dosimetry for ^213^Bi-PSMA-617 TAT in prostate cancer patients. Theoretically, the ratio between the absorbed dose to tumor and dose-limiting organs (kidneys, salivary glands and red marrow) was found sufficient to warrant clinical translation. Nevertheless, the therapeutic index seems clearly inferior compared to that of ^225^Ac-PSMA-617.

It is common practice that the pharmacokinetics of therapeutic radiopharmaceuticals without sufficient inherent imaging capabilities are approximated by using a diagnostic surrogate radionuclide. ^68^Ga is a positron emitter and has the advantage of high resolution, overlap-free tomographic reconstruction and possible quantification by PET. As ^68^Ga has a minimally longer half-life than ^213^Bi, biodistribution data until near total decay of the planned therapeutics can be assessed, avoiding the common challenge that the integration to infinity has to be estimated by curve fitting. Nevertheless, the radio-metal in the chelator complex can theoretically affect the pharmacokinetics of the radiotracer and sometimes these effects are non-negligible [20]. Therefore, using ^68^Ga-PSMA-617 imaging to approximate ^213^Bi-PSMA-617 therapy presents both a major strength and simultaneously a mentionable limitation of the used methodology.

Another limitation is that the dosimetry of ^213^Bi-PSMA-617 has been extrapolated from PET data, but in contrast, the dosimetry of the comparator ^225^Ac-PSMA-617 has been estimated using planar scans—a necessary compromise to cope with its longer half-life. Due to the different physical properties of both radionuclides, the kidney-absorbed dose might be over-estimated for ^213^Bi-PSMA-617. At early time points, there is still activity in cavities of kidney calices/pelvis, contributing counts to the organ VOIs during imaging. But due to the limited penetration range of the alpha particles, activity deposits there would not reach the parenchyma in the renal cortex during TAT. In contrast, images of delayed time points in ^225^Ac-PSMA-617-dosimetry should include a higher fraction of activity representing specific uptake at renal PSMA or due to reabsorption by tubular cells and must be considered more toxically relevant.

Tumor lesion dosimetry is always challenging due to high inter-individual and even inter-lesion variability and should generally be interpreted very cautiously. In addition, the tumor dosimetry estimated for ^225^Ac-PSMA-617 contained remarkable methodical uncertainties because the limited resolution of the underlying planar scans hampers lesion delineation, contrast recovery and suffers from overlap, spill-out and partial volume effects. The a priori assumption that translocation of daughter nuclides can be neglected seems also more prudent for ^213^Bi than for the multi-step decay schema of ^225^Ac. However, the tumor to healthy organ ratios between ^213^Bi-PSMA-617 and ^225^Ac-PSMA-617 are in remarkably different dimensions (Table 2) and the reasons can easily be found by interpretation of the graphic images Figs. 1 and 2. As reported previously, ^225^Ac-PSMA-617 can benefit from continuing renal clearance beyond 5 h p.i. and further accumulation in tumor lesions with maximum uptake approximately 24 h p.i. [5]. Some preliminary case reports are also in line: Two impressive complete remissions could already be observed after ^225^Ac-PSMA-617 [4], but only a case of partial remission following ^213^Bi-PSMA-617 therapy can be found in the literature [12]. Thus, in combination with PSMA-617, ^225^Ac must be considered the first-choice isotope for PSMA-TAT in the setting of prostate cancer.

PSMA expression is also found in the neovasculature of other tumor entities, and especially in clear cell renal carcinoma, PSMA-PET/CT can achieve similar high uptake values as in prostate cancer [21, 22]. However, only the first hours post-injection could be assessed by PSMA-PET. Different uptake kinetics of PSMA ligands in kidneys than in prostate cancer have been reported by two different groups during preclinical experiments [23, 24] and the respective authors discussed that a reduced rate of cellular internalization in renal cells would explain these observations. Until now, tumor retention times of PSMA ligands—either in renal cancer lesions or other tumor entities—have not yet been evaluated systematically. If PSMA on the surface of renal cell cancer is indeed not sufficiently internalized after binding of a ligand, RLT based on a nuclide with multiple instable daughters, such as ^225^Ac, might be suboptimal. Vice versa, vascular expression could further improve target accessibility and accelerate uptake kinetics. We, therefore, should keep in mind that dosimetry of ^213^Bi-PSMA-617 per se allows clinical application; eventually, other clinical indications which could benefit from the specific characteristics of ^213^Bi-PSMA-617 may appear in the future.

## Conclusion

Dosimetry of ^213^Bi-PSMA-617 is in a range traditionally considered promising for clinical application. However, compared to ^225^Ac-PSMA-617, its therapeutic index for therapy of prostate cancer appears to be inferior.

## Electronic supplementary material

ESM 1 (DOCX 70 kb)
