# Supplementary material for: Targeted alpha therapy of mCRPC: Dosimetry estimate of 213Bismuth-PSMA-617
Source: Eur J Nucl Med Mol Imaging. 2017 Sep 11;45(1):31–7. doi: 10.1007/s00259-017-3817-y (PMC5700223; doi:10.1007/s00259-017-3817-y)
Supplement: Supplementary file 1 — (DOCX 70 kb) [file 259_2017_3817_MOESM1_ESM.docx]

Supplementary Material

Targeted Alpha Therapy of mCRPC: Dosimetry estimate of ^213^Bismuth-PSMA-617

1. Supplement Table-1: Patient characteristics

| Patient No. | Age (y) | Body weight (kg) | Body height (cm) | Kidneys, sum (ml) | PSA (ng/ml) | Gleason Score | Previous treatment | LN metastases | Bone metastases | Creatinine (mg/dl) | ^68^Ga-PSMA-617 (MBq) |
| --- | --- | --- | --- | --- | --- | --- | --- | --- | --- | --- | --- |
| 1 | 60 | 110 | 180 | 296 | 2.9 | 9 | RT | >10 | >10 | 1.0 | 221 |
| 2 | 67 | 72 | 181 | 334 | 6.6 | 7 | RPx + RT | 2 | 0 | 0.9 | 260 |
| 3 | 75 | 70 | 172 | 440 | 14.1 | 9 | RPx + ADT | >10 | 0 | 0.7 | 230 |

RPx = prostatectomy, RT = radiotherapy, ADT = androgen deprivation therapy

1. Supplement Table-2: Time-Activity-Curves (^213^Bi; i.e. after half-life extrapolation)

| Timepoint [h] | 0,11 | 1,1 | 2,04 | 3,06 | 4,1 | 5,07 |
| --- | --- | --- | --- | --- | --- | --- |
| Kidneys | 1,01E+01 | 7,01E+00 | 3,33E+00 | 1,32E+00 | 4,69E-01 | 1,86E-01 |
| LParotid Gland | 6,33E-01 | 4,49E-01 | 1,81E-01 | 1,06E-01 | 3,77E-02 | 1,58E-02 |
| LSubmandibular Gl | 2,75E-01 | 1,41E-01 | 5,42E-02 | 3,10E-02 | 1,07E-02 | 4,41E-03 |
| Lesion 1 | 3,40E-01 | 2,78E-01 | 1,55E-01 | 5,55E-02 | 2,66E-02 | 1,24E-02 |
| Lesion 2 | 1,09E-01 | 1,01E-01 | 4,77E-02 | 2,40E-02 | 1,11E-02 | 3,39E-03 |
| Lesion 3 | 4,84E-02 | 3,98E-02 | 2,23E-02 | 1,02E-02 | 4,10E-03 | 2,24E-03 |
| Liver | 1,77E+01 | 6,13E+00 | 2,01E+00 | 6,88E-01 | 2,55E-01 | 1,08E-01 |
| RParotid Gland | 9,45E-01 | 5,57E-01 | 2,46E-01 | 1,15E-01 | 4,36E-02 | 1,60E-02 |
| RSubmandibular Gl | 2,87E-01 | 1,37E-01 | 6,93E-02 | 2,81E-02 | 9,89E-03 | 4,87E-03 |
| Spleen | 2,88E+00 | 1,07E+00 | 3,52E-01 | 1,20E-01 | 3,77E-02 | 1,52E-02 |
| Total Body | 1,97E+02 | 7,53E+01 | 3,02E+01 | 1,09E+01 | 3,86E+00 | 1,56E+00 |
|  |  |  |  |  |  |  |
| Timepoint [h] | 1,07 | 2 | 3,02 | 3,95 | 5,04 |  |
| Red Marrow | 1,18E+00 | 3,89E-01 | 1,40E-01 | 4,63E-02 | 1,39E-02 |  |
|  |  |  |  |  |  |  |
| Timepoint [h] | 0,11 | 1,07 | 1,1 | 2 | 2,04 | 3,02 |
| Urinary Bladder | 6,84E-02 | 2,81E+00 | 5,45E-01 | 1,70E+00 | 2,50E-01 | 8,11E-01 |
| Timepoint [h] | 3,06 | 3,95 | 4,1 | 5,04 | 5,07 |  |
| Urinary Bladder | 1,17E-01 | 2,65E-01 | 4,10E-02 | 7,87E-02 | 1,68E-02 |  |
|  |  |  |  |  |  |  |
| Timepoint [h] | 0,07 | 0,99 | 1,99 | 3,05 | 4,02 | 5 |
| Kidneys | 1,19E+01 | 1,16E+01 | 5,47E+00 | 2,26E+00 | 8,23E-01 | 3,24E-01 |
| LParotid Gland | 1,18E+00 | 7,87E-01 | 4,17E-01 | 1,85E-01 | 6,84E-02 | 3,09E-02 |
| LSubmandibular Gl | 5,12E-01 | 3,62E-01 | 1,37E-01 | 7,09E-02 | 3,10E-02 | 1,07E-02 |
| Lesion 1 | 4,75E-02 | 3,06E-02 | 1,44E-02 | 7,64E-03 | 3,72E-03 | 1,38E-03 |
| Lesion 2 | 5,24E-02 | 4,04E-02 | 2,02E-02 | 9,27E-03 | 4,42E-03 | 1,45E-03 |
| Liver | 2,04E+01 | 6,56E+00 | 2,27E+00 | 8,38E-01 | 3,00E-01 | 1,04E-01 |
| RParotid Gland | 1,37E+00 | 8,68E-01 | 4,58E-01 | 1,86E-01 | 8,04E-02 | 3,16E-02 |
| RSubmandibular Gl | 5,27E-01 | 3,27E-01 | 1,49E-01 | 6,27E-02 | 2,86E-02 | 1,08E-02 |
| Spleen | 7,60E-01 | 2,87E-01 | 1,02E-01 | 3,33E-02 | 1,16E-02 | 4,01E-03 |
| Total Body | 2,00E+02 | 8,30E+01 | 3,22E+01 | 1,18E+01 | 4,64E+00 | 1,85E+00 |
|  |  |  |  |  |  |  |
| Timepoint [h] | 0,97 | 1,97 | 3,02 | 4 | 5 |  |
| Red Marrow | 1,65E+00 | 4,93E-01 | 1,57E-01 | 5,63E-02 | 1,92E-02 |  |
|  |  |  |  |  |  |  |
| Timepoint [h] | 0,07 | 0,97 | 0,99 | 1,97 | 1,99 | 3,02 |
| Urinary Bladder | 0,00E+00 | 4,82E-01 | 4,96E-01 | 5,64E-01 | 8,48E-01 | 9,07E-01 |
| Timepoint [h] | 3,05 | 4 | 4,02 | 5 | 5 |  |
| Urinary Bladder | 1,67E-01 | 2,95E-01 | 4,91E-02 | 1,84E-02 | 1,04E-01 |  |
|  |  |  |  |  |  |  |
| Timepoint [h] | 0,3 | 1,22 | 2,17 | 3,25 | 4,24 | 4,88 |
| Kidneys | 1,07E+01 | 9,95E+00 | 5,11E+00 | 1,84E+00 | 7,33E-01 | 3,12E-01 |
| LParotid Gland | 8,17E-01 | 4,07E-01 | 2,38E-01 | 8,87E-02 | 3,71E-02 | 0,00E+00 |
| LSubmandibular Gl | 3,09E-01 | 1,68E-01 | 8,91E-02 | 3,77E-02 | 1,51E-02 | 0,00E+00 |
| Lesion 1 | 3,39E-02 | 2,06E-02 | 1,17E-02 | 5,30E-03 | 1,49E-03 | 6,44E-04 |
| Liver | 2,20E+01 | 8,41E+00 | 3,09E+00 | 1,02E+00 | 4,07E-01 | 1,65E-01 |
| RParotid Gland | 8,70E-01 | 6,14E-01 | 2,66E-01 | 1,15E-01 | 4,68E-02 | 0,00E+00 |
| RSubmandibular Gl | 2,91E-01 | 1,79E-01 | 7,65E-02 | 3,81E-02 | 1,65E-02 | 0,00E+00 |
| Spleen | 2,72E+00 | 1,23E+00 | 4,87E-01 | 1,37E-01 | 5,77E-02 | 1,83E-02 |
| Total Body | 1,89E+02 | 7,94E+01 | 3,26E+01 | 1,15E+01 | 4,54E+00 | 2,35E+00 |

1. Supplement Table-3: Cumulated Activity (AHat, MBq*h), ResidenceTimes
   (ResTime, MBq*h/MBq), Biological and Effective half-lifes (h)

|  | Ahat | ResTime | Biol. T1/2 | Eff. T1/2 |
| --- | --- | --- | --- | --- |
|  | MBq*h | MBq*h/MBq | h | h |
|  |  |  |  |  |
| Kidneys | 1,77E+01 | 8,00E-02 | 9,04E+00 | 7,01E-01 |
| LParotid Gland | 1,13E+00 | 5,10E-03 | 1,11E+01 | 7,11E-01 |
| LSubmandibular Gland | 3,89E-01 | 1,76E-03 | 7,96E+00 | 6,94E-01 |
| Lesion 1 | 7,14E-01 | 3,23E-03 | -3,78E+00 | 9,51E-01 |
| Lesion 2 | 2,47E-01 | 1,12E-03 | -9,35E+00 | 8,27E-01 |
| Lesion 3 | 1,05E-01 | 4,74E-04 | -6,96E+00 | 8,53E-01 |
| Liver | 1,87E+01 | 8,46E-02 | 3,85E+00 | 6,35E-01 |
| RParotid Gland | 1,49E+00 | 6,72E-03 | 1,68E+01 | 7,27E-01 |
| RSubmandibular Gland | 4,03E-01 | 1,82E-03 | 2,22E+01 | 7,35E-01 |
| Red Marrow | 1,76E+00 | 7,96E-03 | 2,88E+00 | 6,01E-01 |
| Spleen | 3,14E+00 | 1,42E-02 | 2,85E+00 | 6,00E-01 |
| Total Body | 2,28E+02 | 1,03E+00 | 1,12E+01 | 7,12E-01 |
| Urinary Bladder Content | 3,29E+00 | 1,49E-02 | -7,53E+00 | 8,45E-01 |
|  |  |  |  |  |
| Kidneys | 2,63E+01 | 1,23E-01 | 6,25E+00 | 6,77E-01 |
| LParotid Gland | 2,07E+00 | 9,70E-03 | 1,08E+01 | 7,10E-01 |
| LSubmandibular Gland | 8,64E-01 | 4,06E-03 | -4,81E+01 | 7,72E-01 |
| Lesion 1 | 8,20E-02 | 3,85E-04 | -4,59E+00 | 9,10E-01 |
| Lesion 2 | 1,02E-01 | 4,79E-04 | -8,40E+00 | 8,35E-01 |
| Liver | 2,00E+01 | 9,41E-02 | 5,35E+00 | 6,65E-01 |
| RParotid Gland | 2,31E+00 | 1,08E-02 | -2,21E+01 | 7,87E-01 |
| RSubmandibular Gland | 8,40E-01 | 3,94E-03 | -1,08E+01 | 8,17E-01 |
| Red Marrow | 2,37E+00 | 1,11E-02 | 4,14E+00 | 6,42E-01 |
| Spleen | 8,09E-01 | 3,80E-03 | 5,06E+00 | 6,61E-01 |
| Total Body | 2,31E+02 | 1,09E+00 | 1,87E+01 | 7,30E-01 |
| Urinary Bladder Content | 2,05E+00 | 9,63E-03 | -8,67E-01 | 6,16E+00 |
|  |  |  |  |  |
| Kidneys | 2,40E+01 | 9,47E-02 | 8,45E+00 | 6,97E-01 |
| LParotid Gland | 1,27E+00 | 5,02E-03 | -1,72E+02 | 7,63E-01 |
| LSubmandibular Gland | 5,02E-01 | 1,98E-03 | -7,71E+00 | 8,43E-01 |
| Lesion 1 | 5,98E-02 | 2,36E-04 | 1,14E+02 | 7,55E-01 |
| Liver | 2,60E+01 | 1,03E-01 | 5,08E+00 | 6,61E-01 |
| RParotid Gland | 1,58E+00 | 6,24E-03 | -6,44E+00 | 8,61E-01 |
| RSubmandibular Gland | 4,96E-01 | 1,96E-03 | -3,16E+00 | 1,00E+00 |
| Spleen | 3,53E+00 | 1,39E-02 | 6,50E+00 | 6,80E-01 |
| Total Body | 2,42E+02 | 9,55E-01 | 2,02E+01 | 7,32E-01 |

1. Supplement Table-4: OLINDA Interim Results
   (Sv/GBq; Radiation Weighting Factors: Alpha=5, Beta=1, Photon=1; Phantom: Adult Male)

Patient-1

|  | **Bi-213** |  |  |  |  | **Tl-209** |  |  |  |
| --- | --- | --- | --- | --- | --- | --- | --- | --- | --- |
| Target Organ | Alpha | Beta | Photon | Total |  | Alpha | Beta | Photon | Total |
| Adrenals | 3,55E-03 | 2,58E-03 | 7,11E-04 | 6,85E-03 |  | 0,00E+00 | 3,96E-03 | 1,07E-02 | 1,47E-02 |
| Brain | 3,55E-03 | 2,58E-03 | 2,35E-04 | 6,37E-03 |  | 0,00E+00 | 3,96E-03 | 3,57E-03 | 7,53E-03 |
| Breasts | 3,55E-03 | 2,58E-03 | 2,34E-04 | 6,37E-03 |  | 0,00E+00 | 3,96E-03 | 3,74E-03 | 7,70E-03 |
| Gallbladder Wall | 3,55E-03 | 2,58E-03 | 7,33E-04 | 6,87E-03 |  | 0,00E+00 | 3,96E-03 | 1,08E-02 | 1,48E-02 |
| LLI Wall | 3,55E-03 | 2,58E-03 | 3,91E-04 | 6,53E-03 |  | 0,00E+00 | 3,96E-03 | 5,99E-03 | 9,95E-03 |
| Small Intestine | 3,55E-03 | 2,58E-03 | 4,78E-04 | 6,61E-03 |  | 0,00E+00 | 3,96E-03 | 7,22E-03 | 1,12E-02 |
| Stomach Wall | 3,55E-03 | 2,58E-03 | 4,87E-04 | 6,62E-03 |  | 0,00E+00 | 3,96E-03 | 7,34E-03 | 1,13E-02 |
| ULI Wall | 3,55E-03 | 2,58E-03 | 4,84E-04 | 6,62E-03 |  | 0,00E+00 | 3,96E-03 | 7,33E-03 | 1,13E-02 |
| Heart Wall | 3,55E-03 | 2,58E-03 | 4,30E-04 | 6,57E-03 |  | 0,00E+00 | 3,96E-03 | 6,58E-03 | 1,05E-02 |
| Kidneys | 1,12E-01 | 8,05E-02 | 2,13E-03 | 1,94E-01 |  | 0,00E+00 | 1,23E-01 | 3,15E-02 | 1,55E-01 |
| Liver | 1,90E-02 | 1,38E-02 | 9,54E-04 | 3,37E-02 |  | 0,00E+00 | 2,12E-02 | 1,42E-02 | 3,54E-02 |
| Lungs | 3,55E-03 | 2,58E-03 | 3,63E-04 | 6,50E-03 |  | 0,00E+00 | 3,96E-03 | 5,53E-03 | 9,50E-03 |
| Muscle | 3,55E-03 | 2,58E-03 | 3,29E-04 | 6,47E-03 |  | 0,00E+00 | 3,96E-03 | 5,10E-03 | 9,06E-03 |
| Ovaries | 3,55E-03 | 2,58E-03 | 4,21E-04 | 6,56E-03 |  | 0,00E+00 | 3,96E-03 | 6,36E-03 | 1,03E-02 |
| Pancreas | 3,55E-03 | 2,58E-03 | 6,85E-04 | 6,82E-03 |  | 0,00E+00 | 3,96E-03 | 1,00E-02 | 1,40E-02 |
| Red Marrow | 5,14E-03 | 1,82E-03 | 3,91E-04 | 7,36E-03 |  | 0,00E+00 | 2,79E-03 | 5,81E-03 | 8,60E-03 |
| Osteogenic Cells | 5,26E-02 | 4,46E-03 | 4,17E-04 | 5,75E-02 |  | 0,00E+00 | 7,01E-03 | 6,13E-03 | 1,31E-02 |
| Skin | 3,55E-03 | 2,58E-03 | 2,07E-04 | 6,34E-03 |  | 0,00E+00 | 3,96E-03 | 3,28E-03 | 7,24E-03 |
| Spleen | 2,68E-02 | 1,93E-02 | 9,52E-04 | 4,71E-02 |  | 0,00E+00 | 2,95E-02 | 1,42E-02 | 4,37E-02 |
| Testes | 3,55E-03 | 2,58E-03 | 2,74E-04 | 6,41E-03 |  | 0,00E+00 | 3,96E-03 | 4,27E-03 | 8,23E-03 |
| Thymus | 3,55E-03 | 2,58E-03 | 3,23E-04 | 6,46E-03 |  | 0,00E+00 | 3,96E-03 | 4,99E-03 | 8,95E-03 |
| Thyroid | 3,55E-03 | 2,58E-03 | 3,03E-04 | 6,44E-03 |  | 0,00E+00 | 3,96E-03 | 4,64E-03 | 8,60E-03 |
| Urinary Bladder Wall | 3,55E-03 | 2,58E-03 | 3,72E-04 | 6,51E-03 |  | 0,00E+00 | 3,96E-03 | 5,69E-03 | 9,65E-03 |
| Uterus | 3,55E-03 | 2,58E-03 | 4,23E-04 | 6,56E-03 |  | 0,00E+00 | 3,96E-03 | 6,43E-03 | 1,04E-02 |
| Total Body | 4,57E-03 | 3,32E-03 | 3,54E-04 | 8,24E-03 |  | 0,00E+00 | 5,09E-03 | 5,43E-03 | 1,05E-02 |
|  |  |  |  |  |  |  |  |  |  |
|  | **Po-213** |  |  |  |  | **Pb-209** |  |  |  |
| Target Organ | Alpha | Beta | Photon | Total |  | Alpha | Beta | Photon | Total |
| Adrenals | 2,44E-01 | 0,00E+00 | 0,00E+00 | 2,44E-01 |  | 0,00E+00 | 1,15E-03 | 0,00E+00 | 1,15E-03 |
| Brain | 2,44E-01 | 0,00E+00 | 0,00E+00 | 2,44E-01 |  | 0,00E+00 | 1,15E-03 | 0,00E+00 | 1,15E-03 |
| Breasts | 2,44E-01 | 0,00E+00 | 0,00E+00 | 2,44E-01 |  | 0,00E+00 | 1,15E-03 | 0,00E+00 | 1,15E-03 |
| Gallbladder Wall | 2,44E-01 | 0,00E+00 | 0,00E+00 | 2,44E-01 |  | 0,00E+00 | 1,15E-03 | 0,00E+00 | 1,15E-03 |
| LLI Wall | 2,44E-01 | 0,00E+00 | 0,00E+00 | 2,44E-01 |  | 0,00E+00 | 1,15E-03 | 0,00E+00 | 1,15E-03 |
| Small Intestine | 2,44E-01 | 0,00E+00 | 0,00E+00 | 2,44E-01 |  | 0,00E+00 | 1,15E-03 | 0,00E+00 | 1,15E-03 |
| Stomach Wall | 2,44E-01 | 0,00E+00 | 0,00E+00 | 2,44E-01 |  | 0,00E+00 | 1,15E-03 | 0,00E+00 | 1,15E-03 |
| ULI Wall | 2,44E-01 | 0,00E+00 | 0,00E+00 | 2,44E-01 |  | 0,00E+00 | 1,15E-03 | 0,00E+00 | 1,15E-03 |
| Heart Wall | 2,44E-01 | 0,00E+00 | 0,00E+00 | 2,44E-01 |  | 0,00E+00 | 1,15E-03 | 0,00E+00 | 1,15E-03 |
| Kidneys | 7,65E+00 | 0,00E+00 | 0,00E+00 | 7,65E+00 |  | 0,00E+00 | 3,61E-02 | 0,00E+00 | 3,61E-02 |
| Liver | 1,30E+00 | 0,00E+00 | 0,00E+00 | 1,30E+00 |  | 0,00E+00 | 6,14E-03 | 0,00E+00 | 6,14E-03 |
| Lungs | 2,44E-01 | 0,00E+00 | 0,00E+00 | 2,44E-01 |  | 0,00E+00 | 1,15E-03 | 0,00E+00 | 1,15E-03 |
| Muscle | 2,44E-01 | 0,00E+00 | 0,00E+00 | 2,44E-01 |  | 0,00E+00 | 1,15E-03 | 0,00E+00 | 1,15E-03 |
| Ovaries | 2,44E-01 | 0,00E+00 | 0,00E+00 | 2,44E-01 |  | 0,00E+00 | 1,15E-03 | 0,00E+00 | 1,15E-03 |
| Pancreas | 2,44E-01 | 0,00E+00 | 0,00E+00 | 2,44E-01 |  | 0,00E+00 | 1,15E-03 | 0,00E+00 | 1,15E-03 |
| Red Marrow | 3,52E-01 | 0,00E+00 | 0,00E+00 | 3,52E-01 |  | 0,00E+00 | 8,15E-04 | 0,00E+00 | 8,15E-04 |
| Osteogenic Cells | 2,68E+00 | 0,00E+00 | 0,00E+00 | 2,68E+00 |  | 0,00E+00 | 2,71E-03 | 0,00E+00 | 2,71E-03 |
| Skin | 2,44E-01 | 0,00E+00 | 0,00E+00 | 2,44E-01 |  | 0,00E+00 | 1,15E-03 | 0,00E+00 | 1,15E-03 |
| Spleen | 1,84E+00 | 0,00E+00 | 0,00E+00 | 1,84E+00 |  | 0,00E+00 | 8,68E-03 | 0,00E+00 | 8,68E-03 |
| Testes | 2,44E-01 | 0,00E+00 | 0,00E+00 | 2,44E-01 |  | 0,00E+00 | 1,15E-03 | 0,00E+00 | 1,15E-03 |
| Thymus | 2,44E-01 | 0,00E+00 | 0,00E+00 | 2,44E-01 |  | 0,00E+00 | 1,15E-03 | 0,00E+00 | 1,15E-03 |
| Thyroid | 2,44E-01 | 0,00E+00 | 0,00E+00 | 2,44E-01 |  | 0,00E+00 | 1,15E-03 | 0,00E+00 | 1,15E-03 |
| Urinary Bladder Wall | 2,44E-01 | 0,00E+00 | 0,00E+00 | 2,44E-01 |  | 0,00E+00 | 1,15E-03 | 0,00E+00 | 1,15E-03 |
| Uterus | 2,44E-01 | 0,00E+00 | 0,00E+00 | 2,44E-01 |  | 0,00E+00 | 1,15E-03 | 0,00E+00 | 1,15E-03 |
| Total Body | 3,13E-01 | 0,00E+00 | 0,00E+00 | 3,13E-01 |  | 0,00E+00 | 1,48E-03 | 0,00E+00 | 1,48E-03 |
|  |  |  |  |  |  |  |  |  |  |
| **Bi-213 + (0.98*Pb-213) + (0.02*Tl-209) + Pb-209** | | | | |  |  |  |  |  |
| Target Organ | Alpha | Beta | Photon | Total |  | Alpha | Beta | Photon |  |
| Adrenals | 2,43E-01 | 3,81E-03 | 9,25E-04 | 2,47E-01 |  | 98,08 | 1,54 | 0,37 | % |
| Brain | 2,43E-01 | 3,81E-03 | 3,06E-04 | 2,47E-01 |  | 98,33 | 1,54 | 0,12 | % |
| Breasts | 2,43E-01 | 3,81E-03 | 3,09E-04 | 2,47E-01 |  | 98,33 | 1,54 | 0,13 | % |
| Gallbladder Wall | 2,43E-01 | 3,81E-03 | 9,49E-04 | 2,47E-01 |  | 98,07 | 1,54 | 0,38 | % |
| LLI Wall | 2,43E-01 | 3,81E-03 | 5,11E-04 | 2,47E-01 |  | 98,25 | 1,54 | 0,21 | % |
| Small Intestine | 2,43E-01 | 3,81E-03 | 6,22E-04 | 2,47E-01 |  | 98,21 | 1,54 | 0,25 | % |
| Stomach Wall | 2,43E-01 | 3,81E-03 | 6,34E-04 | 2,47E-01 |  | 98,20 | 1,54 | 0,26 | % |
| ULI Wall | 2,43E-01 | 3,81E-03 | 6,31E-04 | 2,47E-01 |  | 98,20 | 1,54 | 0,26 | % |
| Heart Wall | 2,43E-01 | 3,81E-03 | 5,62E-04 | 2,47E-01 |  | 98,23 | 1,54 | 0,23 | % |
| Kidneys | 7,61E+00 | 1,19E-01 | 2,76E-03 | 7,73E+00 |  | 98,43 | 1,54 | 0,04 | % |
| Liver | 1,29E+00 | 2,04E-02 | 1,24E-03 | 1,31E+00 |  | 98,36 | 1,55 | 0,09 | % |
| Lungs | 2,43E-01 | 3,81E-03 | 4,74E-04 | 2,47E-01 |  | 98,26 | 1,54 | 0,19 | % |
| Muscle | 2,43E-01 | 3,81E-03 | 4,31E-04 | 2,47E-01 |  | 98,28 | 1,54 | 0,17 | % |
| Ovaries | 2,43E-01 | 3,81E-03 | 5,48E-04 | 2,47E-01 |  | 98,23 | 1,54 | 0,22 | % |
| Pancreas | 2,43E-01 | 3,81E-03 | 8,85E-04 | 2,47E-01 |  | 98,10 | 1,54 | 0,36 | % |
| Red Marrow | 3,50E-01 | 2,69E-03 | 5,07E-04 | 3,53E-01 |  | 99,09 | 0,76 | 0,14 | % |
| Osteogenic Cells | 2,68E+00 | 7,31E-03 | 5,40E-04 | 2,69E+00 |  | 99,71 | 0,27 | 0,02 | % |
| Skin | 2,43E-01 | 3,81E-03 | 2,73E-04 | 2,47E-01 |  | 98,34 | 1,54 | 0,11 | % |
| Spleen | 1,83E+00 | 2,86E-02 | 1,24E-03 | 1,86E+00 |  | 98,39 | 1,54 | 0,07 | % |
| Testes | 2,43E-01 | 3,81E-03 | 3,59E-04 | 2,47E-01 |  | 98,31 | 1,54 | 0,15 | % |
| Thymus | 2,43E-01 | 3,81E-03 | 4,23E-04 | 2,47E-01 |  | 98,28 | 1,54 | 0,17 | % |
| Thyroid | 2,43E-01 | 3,81E-03 | 3,96E-04 | 2,47E-01 |  | 98,29 | 1,54 | 0,16 | % |
| Urinary Bladder Wall | 2,43E-01 | 3,81E-03 | 4,86E-04 | 2,47E-01 |  | 98,26 | 1,54 | 0,20 | % |
| Uterus | 2,43E-01 | 3,81E-03 | 5,52E-04 | 2,47E-01 |  | 98,23 | 1,54 | 0,22 | % |
| Total Body | 3,11E-01 | 4,90E-03 | 4,63E-04 | 3,17E-01 |  | 98,31 | 1,55 | 0,15 | % |

Patient-2

|  | **Bi-213** |  |  |  |  | **Tl-209** |  |  |  |
| --- | --- | --- | --- | --- | --- | --- | --- | --- | --- |
| Target Organ | Alpha | Beta | Photon | Total |  | Alpha | Beta | Photon | Total |
| Adrenals | 4,04E-03 | 2,93E-03 | 8,00E-04 | 7,77E-03 |  | 0,00E+00 | 4,50E-03 | 1,20E-02 | 1,65E-02 |
| Brain | 4,04E-03 | 2,93E-03 | 2,71E-04 | 7,24E-03 |  | 0,00E+00 | 4,50E-03 | 4,10E-03 | 8,61E-03 |
| Breasts | 4,04E-03 | 2,93E-03 | 2,62E-04 | 7,23E-03 |  | 0,00E+00 | 4,50E-03 | 4,19E-03 | 8,70E-03 |
| Gallbladder Wall | 4,04E-03 | 2,93E-03 | 7,91E-04 | 7,76E-03 |  | 0,00E+00 | 4,50E-03 | 1,17E-02 | 1,62E-02 |
| LLI Wall | 4,04E-03 | 2,93E-03 | 4,63E-04 | 7,43E-03 |  | 0,00E+00 | 4,50E-03 | 6,87E-03 | 1,14E-02 |
| Small Intestine | 4,04E-03 | 2,93E-03 | 5,52E-04 | 7,52E-03 |  | 0,00E+00 | 4,50E-03 | 8,25E-03 | 1,28E-02 |
| Stomach Wall | 4,04E-03 | 2,93E-03 | 5,33E-04 | 7,50E-03 |  | 0,00E+00 | 4,50E-03 | 8,02E-03 | 1,25E-02 |
| ULI Wall | 4,04E-03 | 2,93E-03 | 5,52E-04 | 7,52E-03 |  | 0,00E+00 | 4,50E-03 | 8,27E-03 | 1,28E-02 |
| Heart Wall | 4,04E-03 | 2,93E-03 | 4,75E-04 | 7,45E-03 |  | 0,00E+00 | 4,50E-03 | 7,26E-03 | 1,18E-02 |
| Kidneys | 1,45E-01 | 1,05E-01 | 2,67E-03 | 2,53E-01 |  | 0,00E+00 | 1,60E-01 | 3,94E-02 | 2,00E-01 |
| Liver | 1,74E-02 | 1,26E-02 | 9,52E-04 | 3,09E-02 |  | 0,00E+00 | 1,94E-02 | 1,42E-02 | 3,36E-02 |
| Lungs | 4,04E-03 | 2,93E-03 | 3,99E-04 | 7,37E-03 |  | 0,00E+00 | 4,50E-03 | 6,08E-03 | 1,06E-02 |
| Muscle | 4,04E-03 | 2,93E-03 | 3,77E-04 | 7,35E-03 |  | 0,00E+00 | 4,50E-03 | 5,78E-03 | 1,03E-02 |
| Ovaries | 4,04E-03 | 2,93E-03 | 4,96E-04 | 7,47E-03 |  | 0,00E+00 | 4,50E-03 | 7,29E-03 | 1,18E-02 |
| Pancreas | 4,04E-03 | 2,93E-03 | 7,32E-04 | 7,70E-03 |  | 0,00E+00 | 4,50E-03 | 1,07E-02 | 1,52E-02 |
| Red Marrow | 9,34E-03 | 3,23E-03 | 4,47E-04 | 1,30E-02 |  | 0,00E+00 | 4,93E-03 | 6,59E-03 | 1,15E-02 |
| Osteogenic Cells | 7,61E-02 | 5,78E-03 | 4,77E-04 | 8,23E-02 |  | 0,00E+00 | 9,04E-03 | 6,98E-03 | 1,60E-02 |
| Skin | 4,04E-03 | 2,93E-03 | 2,37E-04 | 7,21E-03 |  | 0,00E+00 | 4,50E-03 | 3,72E-03 | 8,23E-03 |
| Spleen | 7,32E-03 | 5,27E-03 | 7,14E-04 | 1,33E-02 |  | 0,00E+00 | 8,04E-03 | 1,08E-02 | 1,88E-02 |
| Testes | 4,04E-03 | 2,93E-03 | 3,26E-04 | 7,30E-03 |  | 0,00E+00 | 4,50E-03 | 4,92E-03 | 9,42E-03 |
| Thymus | 4,04E-03 | 2,93E-03 | 3,68E-04 | 7,34E-03 |  | 0,00E+00 | 4,50E-03 | 5,65E-03 | 1,02E-02 |
| Thyroid | 4,04E-03 | 2,93E-03 | 3,49E-04 | 7,32E-03 |  | 0,00E+00 | 4,50E-03 | 5,32E-03 | 9,83E-03 |
| Urinary Bladder Wall | 1,21E-02 | 8,78E-03 | 5,61E-04 | 2,14E-02 |  | 0,00E+00 | 4,50E-03 | 6,53E-03 | 1,10E-02 |
| Uterus | 4,04E-03 | 2,93E-03 | 5,14E-04 | 7,49E-03 |  | 0,00E+00 | 4,50E-03 | 7,37E-03 | 1,19E-02 |
| Total Body | 5,15E-03 | 3,74E-03 | 4,01E-04 | 9,30E-03 |  | 0,00E+00 | 5,74E-03 | 6,10E-03 | 1,18E-02 |
|  |  |  |  |  |  |  |  |  |  |
|  | **Po-213** |  |  |  |  | **Pb-209** |  |  |  |
| Target Organ | Alpha | Beta | Photon | Total |  | Alpha | Beta | Photon | Total |
| Adrenals | 2,77E-01 | 0,00E+00 | 0,00E+00 | 2,77E-01 |  | 0,00E+00 | 1,31E-03 | 0,00E+00 | 1,31E-03 |
| Brain | 2,77E-01 | 0,00E+00 | 0,00E+00 | 2,77E-01 |  | 0,00E+00 | 1,31E-03 | 0,00E+00 | 1,31E-03 |
| Breasts | 2,77E-01 | 0,00E+00 | 0,00E+00 | 2,77E-01 |  | 0,00E+00 | 1,31E-03 | 0,00E+00 | 1,31E-03 |
| Gallbladder Wall | 2,77E-01 | 0,00E+00 | 0,00E+00 | 2,77E-01 |  | 0,00E+00 | 1,31E-03 | 0,00E+00 | 1,31E-03 |
| LLI Wall | 2,77E-01 | 0,00E+00 | 0,00E+00 | 2,77E-01 |  | 0,00E+00 | 1,31E-03 | 0,00E+00 | 1,31E-03 |
| Small Intestine | 2,77E-01 | 0,00E+00 | 0,00E+00 | 2,77E-01 |  | 0,00E+00 | 1,31E-03 | 0,00E+00 | 1,31E-03 |
| Stomach Wall | 2,77E-01 | 0,00E+00 | 0,00E+00 | 2,77E-01 |  | 0,00E+00 | 1,31E-03 | 0,00E+00 | 1,31E-03 |
| ULI Wall | 2,77E-01 | 0,00E+00 | 0,00E+00 | 2,77E-01 |  | 0,00E+00 | 1,31E-03 | 0,00E+00 | 1,31E-03 |
| Heart Wall | 2,77E-01 | 0,00E+00 | 0,00E+00 | 2,77E-01 |  | 0,00E+00 | 1,31E-03 | 0,00E+00 | 1,31E-03 |
| Kidneys | 9,96E+00 | 0,00E+00 | 0,00E+00 | 9,96E+00 |  | 0,00E+00 | 4,70E-02 | 0,00E+00 | 4,70E-02 |
| Liver | 1,19E+00 | 0,00E+00 | 0,00E+00 | 1,19E+00 |  | 0,00E+00 | 5,61E-03 | 0,00E+00 | 5,61E-03 |
| Lungs | 2,77E-01 | 0,00E+00 | 0,00E+00 | 2,77E-01 |  | 0,00E+00 | 1,31E-03 | 0,00E+00 | 1,31E-03 |
| Muscle | 2,77E-01 | 0,00E+00 | 0,00E+00 | 2,77E-01 |  | 0,00E+00 | 1,31E-03 | 0,00E+00 | 1,31E-03 |
| Ovaries | 2,77E-01 | 0,00E+00 | 0,00E+00 | 2,77E-01 |  | 0,00E+00 | 1,31E-03 | 0,00E+00 | 1,31E-03 |
| Pancreas | 2,77E-01 | 0,00E+00 | 0,00E+00 | 2,77E-01 |  | 0,00E+00 | 1,31E-03 | 0,00E+00 | 1,31E-03 |
| Red Marrow | 6,40E-01 | 0,00E+00 | 0,00E+00 | 6,40E-01 |  | 0,00E+00 | 1,49E-03 | 0,00E+00 | 1,49E-03 |
| Osteogenic Cells | 4,16E+00 | 0,00E+00 | 0,00E+00 | 4,16E+00 |  | 0,00E+00 | 3,39E-03 | 0,00E+00 | 3,39E-03 |
| Skin | 2,77E-01 | 0,00E+00 | 0,00E+00 | 2,77E-01 |  | 0,00E+00 | 1,31E-03 | 0,00E+00 | 1,31E-03 |
| Spleen | 5,02E-01 | 0,00E+00 | 0,00E+00 | 5,02E-01 |  | 0,00E+00 | 2,37E-03 | 0,00E+00 | 2,37E-03 |
| Testes | 2,77E-01 | 0,00E+00 | 0,00E+00 | 2,77E-01 |  | 0,00E+00 | 1,31E-03 | 0,00E+00 | 1,31E-03 |
| Thymus | 2,77E-01 | 0,00E+00 | 0,00E+00 | 2,77E-01 |  | 0,00E+00 | 1,31E-03 | 0,00E+00 | 1,31E-03 |
| Thyroid | 2,77E-01 | 0,00E+00 | 0,00E+00 | 2,77E-01 |  | 0,00E+00 | 1,31E-03 | 0,00E+00 | 1,31E-03 |
| Urinary Bladder Wall | 2,77E-01 | 0,00E+00 | 0,00E+00 | 2,77E-01 |  | 0,00E+00 | 1,31E-03 | 0,00E+00 | 1,31E-03 |
| Uterus | 2,77E-01 | 0,00E+00 | 0,00E+00 | 2,77E-01 |  | 0,00E+00 | 1,31E-03 | 0,00E+00 | 1,31E-03 |
| Total Body | 3,53E-01 | 0,00E+00 | 0,00E+00 | 3,53E-01 |  | 0,00E+00 | 1,66E-03 | 0,00E+00 | 1,66E-03 |
|  |  |  |  |  |  |  |  |  |  |
| **Bi-213 + (0.98*Pb-213) + (0.02*Tl-209) + Pb-209** | | | | |  |  |  |  |  |
| Target Organ | Alpha | Beta | Photon | Total |  | Alpha | Beta | Photon |  |
| Adrenals | 2,76E-01 | 4,33E-03 | 1,04E-03 | 2,81E-01 |  | 98,09 | 1,54 | 0,37 | % |
| Brain | 2,76E-01 | 4,33E-03 | 3,53E-04 | 2,80E-01 |  | 98,33 | 1,55 | 0,13 | % |
| Breasts | 2,76E-01 | 4,33E-03 | 3,46E-04 | 2,80E-01 |  | 98,33 | 1,55 | 0,12 | % |
| Gallbladder Wall | 2,76E-01 | 4,33E-03 | 1,03E-03 | 2,81E-01 |  | 98,09 | 1,54 | 0,36 | % |
| LLI Wall | 2,76E-01 | 4,33E-03 | 6,00E-04 | 2,80E-01 |  | 98,24 | 1,54 | 0,21 | % |
| Small Intestine | 2,76E-01 | 4,33E-03 | 7,17E-04 | 2,81E-01 |  | 98,20 | 1,54 | 0,26 | % |
| Stomach Wall | 2,76E-01 | 4,33E-03 | 6,93E-04 | 2,81E-01 |  | 98,21 | 1,54 | 0,25 | % |
| ULI Wall | 2,76E-01 | 4,33E-03 | 7,17E-04 | 2,81E-01 |  | 98,20 | 1,54 | 0,26 | % |
| Heart Wall | 2,76E-01 | 4,33E-03 | 6,20E-04 | 2,80E-01 |  | 98,23 | 1,54 | 0,22 | % |
| Kidneys | 9,91E+00 | 1,55E-01 | 3,46E-03 | 1,01E+01 |  | 98,42 | 1,54 | 0,03 | % |
| Liver | 1,18E+00 | 1,86E-02 | 1,24E-03 | 1,20E+00 |  | 98,36 | 1,55 | 0,10 | % |
| Lungs | 2,76E-01 | 4,33E-03 | 5,21E-04 | 2,80E-01 |  | 98,27 | 1,54 | 0,19 | % |
| Muscle | 2,76E-01 | 4,33E-03 | 4,93E-04 | 2,80E-01 |  | 98,28 | 1,54 | 0,18 | % |
| Ovaries | 2,76E-01 | 4,33E-03 | 6,42E-04 | 2,80E-01 |  | 98,23 | 1,54 | 0,23 | % |
| Pancreas | 2,76E-01 | 4,33E-03 | 9,46E-04 | 2,81E-01 |  | 98,12 | 1,54 | 0,34 | % |
| Red Marrow | 6,37E-01 | 4,82E-03 | 5,79E-04 | 6,42E-01 |  | 99,16 | 0,75 | 0,09 | % |
| Osteogenic Cells | 4,15E+00 | 9,35E-03 | 6,17E-04 | 4,16E+00 |  | 99,76 | 0,22 | 0,01 | % |
| Skin | 2,76E-01 | 4,33E-03 | 3,11E-04 | 2,80E-01 |  | 98,34 | 1,55 | 0,11 | % |
| Spleen | 4,99E-01 | 7,80E-03 | 9,30E-04 | 5,08E-01 |  | 98,28 | 1,54 | 0,18 | % |
| Testes | 2,76E-01 | 4,33E-03 | 4,24E-04 | 2,80E-01 |  | 98,30 | 1,55 | 0,15 | % |
| Thymus | 2,76E-01 | 4,33E-03 | 4,81E-04 | 2,80E-01 |  | 98,28 | 1,54 | 0,17 | % |
| Thyroid | 2,76E-01 | 4,33E-03 | 4,55E-04 | 2,80E-01 |  | 98,29 | 1,54 | 0,16 | % |
| Urinary Bladder Wall | 2,84E-01 | 1,02E-02 | 6,92E-04 | 2,94E-01 |  | 96,32 | 3,46 | 0,23 | % |
| Uterus | 2,76E-01 | 4,33E-03 | 6,61E-04 | 2,80E-01 |  | 98,22 | 1,54 | 0,24 | % |
| Total Body | 3,51E-01 | 5,51E-03 | 5,23E-04 | 3,57E-01 |  | 98,31 | 1,54 | 0,15 | % |

Patient-3

|  | **Bi-213** |  |  |  |  | **Tl-209** |  |  |  |
| --- | --- | --- | --- | --- | --- | --- | --- | --- | --- |
| Target Organ | Alpha | Beta | Photon | Total |  | Alpha | Beta | Photon | Total |
| Adrenals | 3,96E-03 | 2,88E-03 | 6,87E-04 | 7,53E-03 |  | 0,00E+00 | 4,42E-03 | 1,03E-02 | 1,48E-02 |
| Brain | 3,96E-03 | 2,88E-03 | 2,65E-04 | 7,11E-03 |  | 0,00E+00 | 4,42E-03 | 4,01E-03 | 8,43E-03 |
| Breasts | 3,96E-03 | 2,88E-03 | 2,53E-04 | 7,09E-03 |  | 0,00E+00 | 4,42E-03 | 4,02E-03 | 8,44E-03 |
| Gallbladder Wall | 3,96E-03 | 2,88E-03 | 7,05E-04 | 7,55E-03 |  | 0,00E+00 | 4,42E-03 | 1,04E-02 | 1,48E-02 |
| LLI Wall | 3,96E-03 | 2,88E-03 | 4,55E-04 | 7,30E-03 |  | 0,00E+00 | 4,42E-03 | 6,59E-03 | 1,10E-02 |
| Small Intestine | 3,96E-03 | 2,88E-03 | 5,16E-04 | 7,36E-03 |  | 0,00E+00 | 4,42E-03 | 7,63E-03 | 1,21E-02 |
| Stomach Wall | 3,96E-03 | 2,88E-03 | 5,10E-04 | 7,35E-03 |  | 0,00E+00 | 4,42E-03 | 7,65E-03 | 1,21E-02 |
| ULI Wall | 3,96E-03 | 2,88E-03 | 5,14E-04 | 7,36E-03 |  | 0,00E+00 | 4,42E-03 | 7,66E-03 | 1,21E-02 |
| Heart Wall | 3,96E-03 | 2,88E-03 | 4,54E-04 | 7,30E-03 |  | 0,00E+00 | 4,42E-03 | 6,92E-03 | 1,13E-02 |
| Kidneys | 9,42E-02 | 6,80E-02 | 1,87E-03 | 1,64E-01 |  | 0,00E+00 | 1,04E-01 | 2,77E-02 | 1,32E-01 |
| Liver | 1,56E-02 | 1,13E-02 | 8,46E-04 | 2,78E-02 |  | 0,00E+00 | 1,74E-02 | 1,26E-02 | 3,00E-02 |
| Lungs | 3,96E-03 | 2,88E-03 | 3,81E-04 | 7,22E-03 |  | 0,00E+00 | 4,42E-03 | 5,80E-03 | 1,02E-02 |
| Muscle | 3,96E-03 | 2,88E-03 | 3,59E-04 | 7,20E-03 |  | 0,00E+00 | 4,42E-03 | 5,46E-03 | 9,88E-03 |
| Ovaries | 3,96E-03 | 2,88E-03 | 4,83E-04 | 7,32E-03 |  | 0,00E+00 | 4,42E-03 | 6,96E-03 | 1,14E-02 |
| Pancreas | 3,96E-03 | 2,88E-03 | 6,83E-04 | 7,52E-03 |  | 0,00E+00 | 4,42E-03 | 9,98E-03 | 1,44E-02 |
| Red Marrow | 8,24E-03 | 2,86E-03 | 4,11E-04 | 1,15E-02 |  | 0,00E+00 | 4,37E-03 | 6,02E-03 | 1,04E-02 |
| Osteogenic Cells | 7,03E-02 | 5,48E-03 | 4,54E-04 | 7,63E-02 |  | 0,00E+00 | 8,59E-03 | 6,62E-03 | 1,52E-02 |
| Skin | 3,96E-03 | 2,88E-03 | 2,26E-04 | 7,07E-03 |  | 0,00E+00 | 4,42E-03 | 3,54E-03 | 7,96E-03 |
| Spleen | 2,73E-02 | 1,97E-02 | 9,55E-04 | 4,80E-02 |  | 0,00E+00 | 3,00E-02 | 1,43E-02 | 4,43E-02 |
| Testes | 3,96E-03 | 2,88E-03 | 3,25E-04 | 7,17E-03 |  | 0,00E+00 | 4,42E-03 | 4,80E-03 | 9,22E-03 |
| Thymus | 3,96E-03 | 2,88E-03 | 3,57E-04 | 7,20E-03 |  | 0,00E+00 | 4,42E-03 | 5,47E-03 | 9,89E-03 |
| Thyroid | 3,96E-03 | 2,88E-03 | 3,41E-04 | 7,18E-03 |  | 0,00E+00 | 4,42E-03 | 5,19E-03 | 9,61E-03 |
| Urinary Bladder Wall | 1,64E-02 | 1,19E-02 | 6,46E-04 | 2,89E-02 |  | 0,00E+00 | 4,42E-03 | 6,33E-03 | 1,08E-02 |
| Uterus | 3,96E-03 | 2,88E-03 | 5,15E-04 | 7,36E-03 |  | 0,00E+00 | 4,42E-03 | 7,06E-03 | 1,15E-02 |
| Total Body | 4,86E-03 | 3,53E-03 | 3,78E-04 | 8,77E-03 |  | 0,00E+00 | 5,42E-03 | 5,72E-03 | 1,11E-02 |
|  |  |  |  |  |  |  |  |  |  |
|  | **Po-213** |  |  |  |  | **Pb-209** |  |  |  |
| Target Organ | Alpha | Beta | Photon | Total |  | Alpha | Beta | Photon | Total |
| Adrenals | 2,72E-01 | 0,00E+00 | 0,00E+00 | 2,72E-01 |  | 0,00E+00 | 1,28E-03 | 0,00E+00 | 1,28E-03 |
| Brain | 2,72E-01 | 0,00E+00 | 0,00E+00 | 2,72E-01 |  | 0,00E+00 | 1,28E-03 | 0,00E+00 | 1,28E-03 |
| Breasts | 2,72E-01 | 0,00E+00 | 0,00E+00 | 2,72E-01 |  | 0,00E+00 | 1,28E-03 | 0,00E+00 | 1,28E-03 |
| Gallbladder Wall | 2,72E-01 | 0,00E+00 | 0,00E+00 | 2,72E-01 |  | 0,00E+00 | 1,28E-03 | 0,00E+00 | 1,28E-03 |
| LLI Wall | 2,72E-01 | 0,00E+00 | 0,00E+00 | 2,72E-01 |  | 0,00E+00 | 1,28E-03 | 0,00E+00 | 1,28E-03 |
| Small Intestine | 2,72E-01 | 0,00E+00 | 0,00E+00 | 2,72E-01 |  | 0,00E+00 | 1,28E-03 | 0,00E+00 | 1,28E-03 |
| Stomach Wall | 2,72E-01 | 0,00E+00 | 0,00E+00 | 2,72E-01 |  | 0,00E+00 | 1,28E-03 | 0,00E+00 | 1,28E-03 |
| ULI Wall | 2,72E-01 | 0,00E+00 | 0,00E+00 | 2,72E-01 |  | 0,00E+00 | 1,28E-03 | 0,00E+00 | 1,28E-03 |
| Heart Wall | 2,72E-01 | 0,00E+00 | 0,00E+00 | 2,72E-01 |  | 0,00E+00 | 1,28E-03 | 0,00E+00 | 1,28E-03 |
| Kidneys | 6,46E+00 | 0,00E+00 | 0,00E+00 | 6,46E+00 |  | 0,00E+00 | 3,05E-02 | 0,00E+00 | 3,05E-02 |
| Liver | 1,07E+00 | 0,00E+00 | 0,00E+00 | 1,07E+00 |  | 0,00E+00 | 5,04E-03 | 0,00E+00 | 5,04E-03 |
| Lungs | 2,72E-01 | 0,00E+00 | 0,00E+00 | 2,72E-01 |  | 0,00E+00 | 1,28E-03 | 0,00E+00 | 1,28E-03 |
| Muscle | 2,72E-01 | 0,00E+00 | 0,00E+00 | 2,72E-01 |  | 0,00E+00 | 1,28E-03 | 0,00E+00 | 1,28E-03 |
| Ovaries | 2,72E-01 | 0,00E+00 | 0,00E+00 | 2,72E-01 |  | 0,00E+00 | 1,28E-03 | 0,00E+00 | 1,28E-03 |
| Pancreas | 2,72E-01 | 0,00E+00 | 0,00E+00 | 2,72E-01 |  | 0,00E+00 | 1,28E-03 | 0,00E+00 | 1,28E-03 |
| Red Marrow | 5,65E-01 | 0,00E+00 | 0,00E+00 | 5,65E-01 |  | 0,00E+00 | 1,31E-03 | 0,00E+00 | 1,31E-03 |
| Osteogenic Cells | 3,79E+00 | 0,00E+00 | 0,00E+00 | 3,79E+00 |  | 0,00E+00 | 3,24E-03 | 0,00E+00 | 3,24E-03 |
| Skin | 2,72E-01 | 0,00E+00 | 0,00E+00 | 2,72E-01 |  | 0,00E+00 | 1,28E-03 | 0,00E+00 | 1,28E-03 |
| Spleen | 1,87E+00 | 0,00E+00 | 0,00E+00 | 1,87E+00 |  | 0,00E+00 | 8,83E-03 | 0,00E+00 | 8,83E-03 |
| Testes | 2,72E-01 | 0,00E+00 | 0,00E+00 | 2,72E-01 |  | 0,00E+00 | 1,28E-03 | 0,00E+00 | 1,28E-03 |
| Thymus | 2,72E-01 | 0,00E+00 | 0,00E+00 | 2,72E-01 |  | 0,00E+00 | 1,28E-03 | 0,00E+00 | 1,28E-03 |
| Thyroid | 2,72E-01 | 0,00E+00 | 0,00E+00 | 2,72E-01 |  | 0,00E+00 | 1,28E-03 | 0,00E+00 | 1,28E-03 |
| Urinary Bladder Wall | 2,72E-01 | 0,00E+00 | 0,00E+00 | 2,72E-01 |  | 0,00E+00 | 1,28E-03 | 0,00E+00 | 1,28E-03 |
| Uterus | 2,72E-01 | 0,00E+00 | 0,00E+00 | 2,72E-01 |  | 0,00E+00 | 1,28E-03 | 0,00E+00 | 1,28E-03 |
| Total Body | 3,33E-01 | 0,00E+00 | 0,00E+00 | 3,33E-01 |  | 0,00E+00 | 1,57E-03 | 0,00E+00 | 1,57E-03 |
|  |  |  |  |  |  |  |  |  |  |
| **Bi-213 + (0.98*Pb-213) + (0.02*Tl-209) + Pb-209** | | | | |  |  |  |  |  |
| Target Organ | Alpha | Beta | Photon | Total |  | Alpha | Beta | Photon |  |
| Adrenals | 2,71E-01 | 4,25E-03 | 8,93E-04 | 2,76E-01 |  | 98,13 | 1,54 | 0,32 | % |
| Brain | 2,71E-01 | 4,25E-03 | 3,45E-04 | 2,75E-01 |  | 98,33 | 1,54 | 0,13 | % |
| Breasts | 2,71E-01 | 4,25E-03 | 3,33E-04 | 2,75E-01 |  | 98,34 | 1,54 | 0,12 | % |
| Gallbladder Wall | 2,71E-01 | 4,25E-03 | 9,13E-04 | 2,76E-01 |  | 98,13 | 1,54 | 0,33 | % |
| LLI Wall | 2,71E-01 | 4,25E-03 | 5,87E-04 | 2,75E-01 |  | 98,24 | 1,54 | 0,21 | % |
| Small Intestine | 2,71E-01 | 4,25E-03 | 6,69E-04 | 2,75E-01 |  | 98,21 | 1,54 | 0,24 | % |
| Stomach Wall | 2,71E-01 | 4,25E-03 | 6,63E-04 | 2,75E-01 |  | 98,22 | 1,54 | 0,24 | % |
| ULI Wall | 2,71E-01 | 4,25E-03 | 6,67E-04 | 2,75E-01 |  | 98,21 | 1,54 | 0,24 | % |
| Heart Wall | 2,71E-01 | 4,25E-03 | 5,92E-04 | 2,75E-01 |  | 98,24 | 1,54 | 0,22 | % |
| Kidneys | 6,43E+00 | 1,01E-01 | 2,42E-03 | 6,53E+00 |  | 98,42 | 1,54 | 0,04 | % |
| Liver | 1,06E+00 | 1,67E-02 | 1,10E-03 | 1,08E+00 |  | 98,35 | 1,54 | 0,10 | % |
| Lungs | 2,71E-01 | 4,25E-03 | 4,97E-04 | 2,75E-01 |  | 98,28 | 1,54 | 0,18 | % |
| Muscle | 2,71E-01 | 4,25E-03 | 4,68E-04 | 2,75E-01 |  | 98,29 | 1,54 | 0,17 | % |
| Ovaries | 2,71E-01 | 4,25E-03 | 6,22E-04 | 2,75E-01 |  | 98,23 | 1,54 | 0,23 | % |
| Pancreas | 2,71E-01 | 4,25E-03 | 8,83E-04 | 2,76E-01 |  | 98,14 | 1,54 | 0,32 | % |
| Red Marrow | 5,62E-01 | 4,26E-03 | 5,31E-04 | 5,67E-01 |  | 99,16 | 0,75 | 0,09 | % |
| Osteogenic Cells | 3,78E+00 | 8,89E-03 | 5,86E-04 | 3,79E+00 |  | 99,75 | 0,23 | 0,02 | % |
| Skin | 2,71E-01 | 4,25E-03 | 2,97E-04 | 2,75E-01 |  | 98,35 | 1,54 | 0,11 | % |
| Spleen | 1,86E+00 | 2,91E-02 | 1,24E-03 | 1,89E+00 |  | 98,39 | 1,54 | 0,07 | % |
| Testes | 2,71E-01 | 4,25E-03 | 4,21E-04 | 2,75E-01 |  | 98,30 | 1,54 | 0,15 | % |
| Thymus | 2,71E-01 | 4,25E-03 | 4,66E-04 | 2,75E-01 |  | 98,29 | 1,54 | 0,17 | % |
| Thyroid | 2,71E-01 | 4,25E-03 | 4,45E-04 | 2,75E-01 |  | 98,30 | 1,54 | 0,16 | % |
| Urinary Bladder Wall | 2,83E-01 | 1,33E-02 | 7,73E-04 | 2,97E-01 |  | 95,29 | 4,47 | 0,26 | % |
| Uterus | 2,71E-01 | 4,25E-03 | 6,56E-04 | 2,75E-01 |  | 98,22 | 1,54 | 0,24 | % |
| Total Body | 3,31E-01 | 5,21E-03 | 4,92E-04 | 3,37E-01 |  | 98,31 | 1,55 | 0,15 | % |

1. Supplement Table-5: SphericalModel, Interim results

|  |  | Method | measured | PowFncFit | PowFncFit | PowFncFit | PowFncFit |  |
| --- | --- | --- | --- | --- | --- | --- | --- | --- |
| Organ / %ID | "res-time" | Unit | ml | mGy/MBq | mGy/MBq | mGy/MBq | mGy/MBq | **Sv_RBE5_/GBq** |
|  | MBqh/MBq | Nuclide |  | Bi-213 | Tl-209 | Po-213 | Pb-209 | **SUM** |
|  |  | Fraction |  | 1 | 0,02 | 0,98 | 1 |  |
|  |  | Weight |  | 1,08 | 1 | 5 | 1 |  |
| Patient-1: |  |  |  |  |  |  |  |  |
| L-Parotid Gland | 0,0050 |  | 20,7 | 0,077 | 0,099 | 1,172 | 0,027 | **5,85** |
| R-Parotid Gland | 0,0062 |  | 20,2 | 0,098 | 0,126 | 1,493 | 0,035 | **7,46** |
| L-Submandibular Gland | 0,0020 |  | 7,2 | 0,086 | 0,105 | 1,329 | 0,031 | **6,64** |
| R-Submandibular Gland | 0,0062 |  | 7,6 | 0,257 | 0,314 | 3,967 | 0,092 | **19,82** |
| Lesion 1 | 0,0002 |  | 1,8 | 0,040 | 0,045 | 0,634 | 0,015 | **3,16** |
| Patient-2: |  |  |  |  |  |  |  |  |
| L-Parotid Gland | 0,0097 |  | 23,7 | 0,130 | 0,169 | 1,978 | 0,046 | **9,88** |
| R-Parotid Gland | 0,0108 |  | 25,9 | 0,133 | 0,174 | 2,015 | 0,047 | **10,07** |
| L-Submandibular Gland | 0,0041 |  | 10,6 | 0,121 | 0,150 | 1,851 | 0,043 | **9,24** |
| R-Submandibular Gland | 0,0039 |  | 9,1 | 0,136 | 0,168 | 2,092 | 0,048 | **10,45** |
| Lesion 1 | 0,0004 |  | 2,9 | 0,041 | 0,048 | 0,642 | 0,015 | **3,20** |
| Lesion 2 | 0,0005 |  | 2,9 | 0,051 | 0,059 | 0,798 | 0,018 | **3,99** |
| Patient-3: |  |  |  |  |  |  |  |  |
| L-Parotid Gland | 0,0051 |  | 38,6 | 0,042 | 0,057 | 0,638 | 0,015 | **3,19** |
| R-Parotid Gland | 0,0067 |  | 37,4 | 0,058 | 0,077 | 0,868 | 0,020 | **4,34** |
| L-Submandibular Gland | 0,0018 |  | 7,6 | 0,073 | 0,089 | 1,119 | 0,026 | **5,59** |
| R-Submandibular Gland | 0,0018 |  | 9,3 | 0,061 | 0,076 | 0,946 | 0,022 | **4,72** |
| Lesion 1 | 0,0032 |  | 10 | 0,102 | 0,126 | 1,561 | 0,036 | **7,80** |
| Lesion 2 | 0,0011 |  | 3 | 0,115 | 0,134 | 1,804 | 0,042 | **9,01** |
| Lesion 3 | 0,0005 |  | 1,5 | 0,097 | 0,108 | 1,527 | 0,035 | **7,62** |
